# Supplementary figures and images for: Prenatal alcohol exposure alters expression of genes involved in cell adhesion, immune response, and toxin metabolism in adolescent rat hippocampus
Source: PLoS One. 2024 Jan 25;19(1):e0293425. doi: 10.1371/journal.pone.0293425 (PMC10810486; doi:10.1371/journal.pone.0293425)

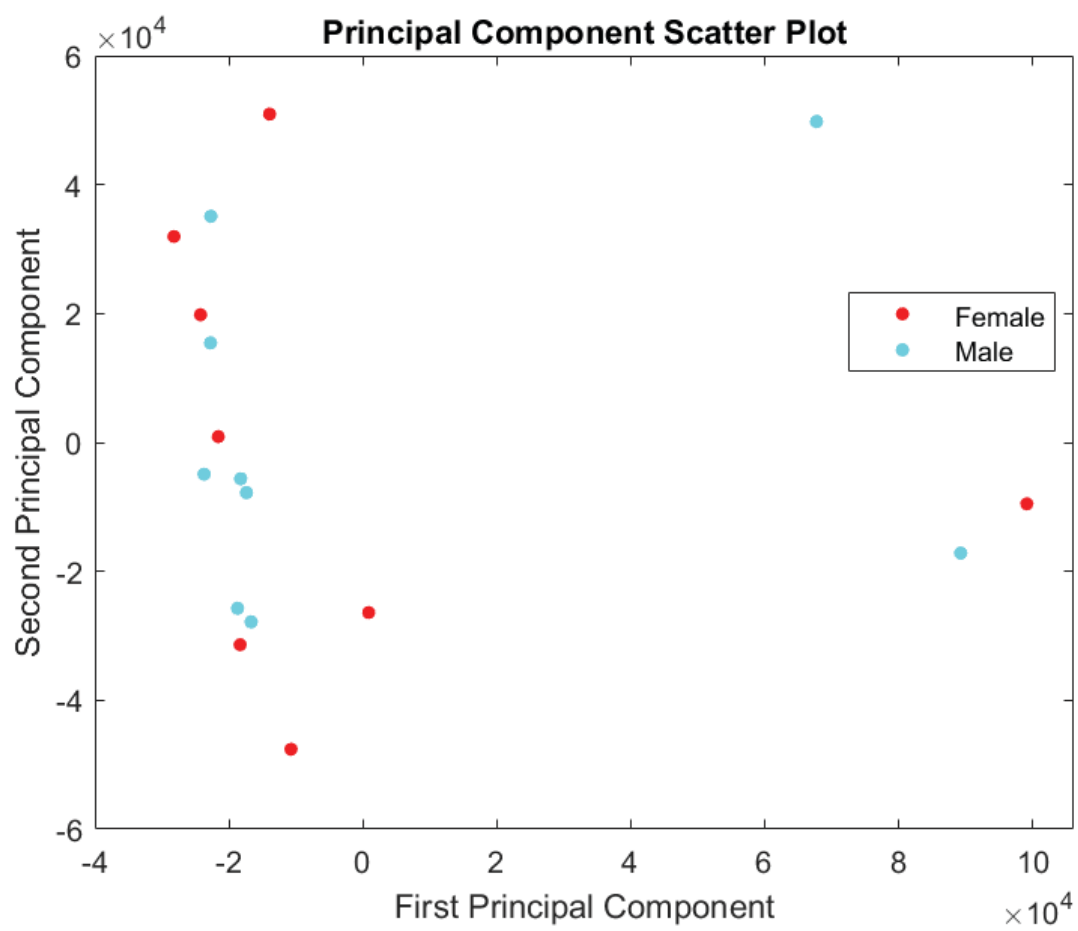

**Supplemental Figure 1**

**A**

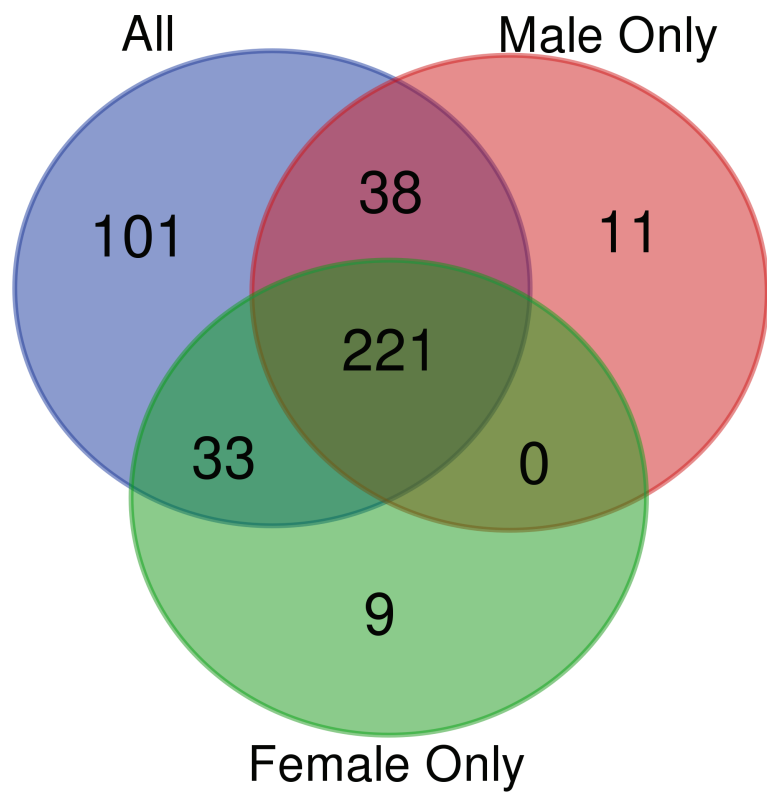

**B**

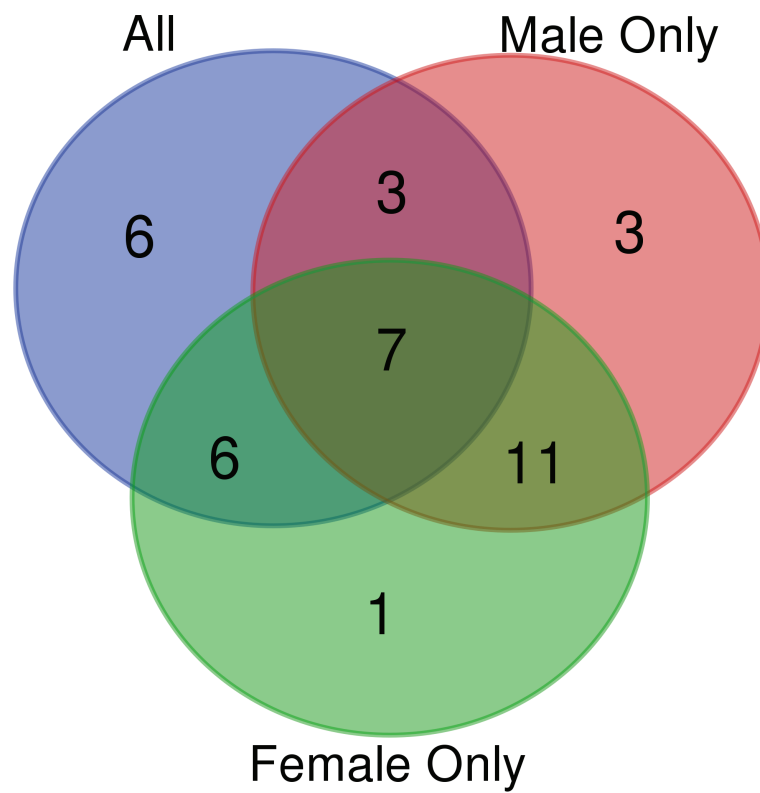

**Supplemental Figure 2**

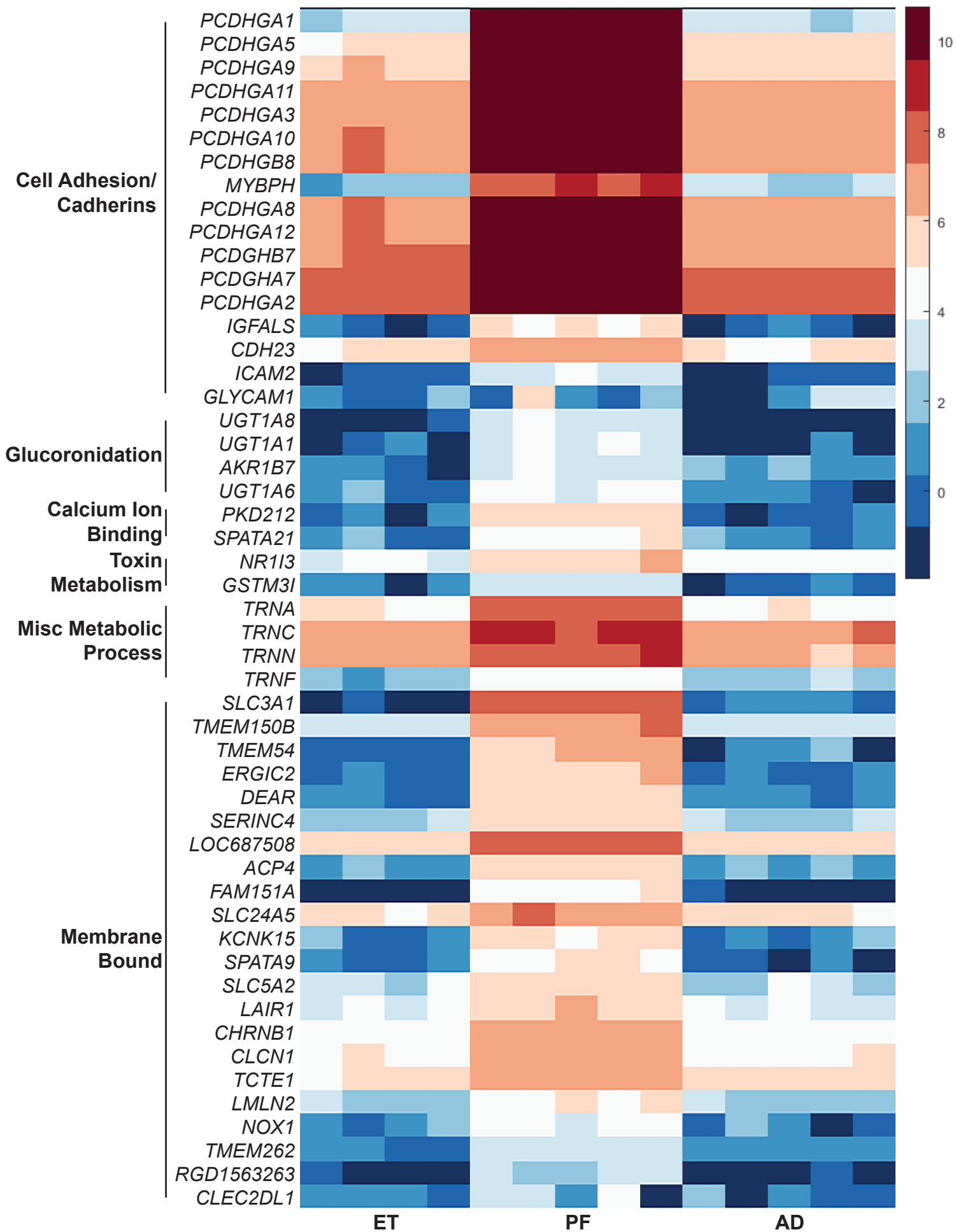

**Supplemental Figure 3**

Supplement: S1 File — (PDF) [file pone.0293425.s001.pdf]
