# Supplementary material for: Prenatal alcohol exposure alters expression of genes involved in cell adhesion, immune response, and toxin metabolism in adolescent rat hippocampus
Source: PLoS One. 2024 Jan 25;19(1):e0293425. doi: 10.1371/journal.pone.0293425 (PMC10810486; doi:10.1371/journal.pone.0293425)
Supplement: Attachment — Submitted filename: Response to Reviewers.docx [file pone.0293425.s004.docx]

PONE-D-23-18670

Response to Reviewers

We thank the reviewers for their insightful comments and have provided a point-by-point response below.

**Comments to the Author**

1. Is the manuscript technically sound, and do the data support the conclusions?

The manuscript must describe a technically sound piece of scientific research with data that supports the conclusions. Experiments must have been conducted rigorously, with appropriate controls, replication, and sample sizes. The conclusions must be drawn appropriately based on the data presented.

Reviewer #1: No

Reviewer #2: Partly

2. Has the statistical analysis been performed appropriately and rigorously?

Reviewer #1: No

Reviewer #2: Yes

3. Have the authors made all data underlying the findings in their manuscript fully available?

The [PLOS Data policy](http://www.plosone.org/static/policies.action#sharing) requires authors to make all data underlying the findings described in their manuscript fully available without restriction, with rare exception (please refer to the Data Availability Statement in the manuscript PDF file). The data should be provided as part of the manuscript or its supporting information, or deposited to a public repository. For example, in addition to summary statistics, the data points behind means, medians and variance measures should be available. If there are restrictions on publicly sharing data—e.g. participant privacy or use of data from a third party—those must be specified.

Reviewer #1: Yes

Reviewer #2: Yes

4. Is the manuscript presented in an intelligible fashion and written in standard English?

PLOS ONE does not copyedit accepted manuscripts, so the language in submitted articles must be clear, correct, and unambiguous. Any typographical or grammatical errors should be corrected at revision, so please note any specific errors here.

Reviewer #1: Yes

Reviewer #2: Yes

5. Review Comments to the Author

Please use the space provided to explain your answers to the questions above. You may also include additional comments for the author, including concerns about dual publication, research ethics, or publication ethics. (Please upload your review as an attachment if it exceeds 20,000 characters)

Reviewer #1: The manuscript titled “Prenatal alcohol exposure alters expression of genes involved in cell adhesion, immune response, and toxin metabolism in adolescent rat hippocampus” by Khalifa and colleagues aimed to characterize the long-term effects of prenatal alcohol exposure on gene expression in rat hippocampus, reporting significant differences in the expression of genes involved in the cell adhesion process and immune response. The current version of the manuscript needs extensive revision before acceptance for publication.

Major comments
• In the introduction section, the authors summarized the current knowledge about prenatal alcohol exposure (PAE) effects, but in the current version, it is not clear the rationale of the present investigation, in particular why they decided to focus their attention on neuroinflammation, G-protein coupled receptor signaling, and cell adhesion. In the last part of the introduction, the authors discussed the data collected to date about the hippocampus and their limitations (sex-variable, age). However, it would be better for validating the importance of the current study to cite previous experimental evidence performed in vitro models (studying the cell-adhesion class) and discuss the importance of alcohol concentration and/or paradigm used, in order to highlight what the present investigation is going to add to the current knowledge.

- *We apologize for the confusion regarding the analyses, but we did not choose to focus on any specific gene targets for the analyses in this manuscript. We conducted an unbiased screen of changes in gene expression and subsequently conducted a gene ontology analysis that revealed significant changes in gene expression in cell adhesion, toxin metabolism, and immune processes. We discuss the relevance of these changes in the discussion.*
- *We have added some references to the introduction and discussion focusing on protocadherins. Importantly, previous studies in humans have shown increased DNA methylation of these genes, lending support to the current work.*
- *We respectfully disagree that a discussion of in vitro models in the introduction is a better justification for the importance of the current study. The current study uses an in vivo model of prenatal alcohol exposure and the literature provided in the introduction highlights findings from studies with similar methodologies. However, we do feel that including a better discussion of the findings of studies measuring cell adhesion is warranted. To this end, we have elected to expand the discussion (Lines 330-355 the revised version) to include these important references. We thank the reviewer for this insightful suggestion and think that these changes greatly improve the discussion.*

• In the same section, the authors reported the current findings about PAE effects on gene expression, but for different studies, they did not report the paradigm used (acute vs long exposure), the alcohol concentration used, voluntary drink vs gavage.

- *We have added some of these details to the current manuscript to address these concerns (Lines 101-120 of revised manuscript).*

• The authors from lines 66 to 69 listed the neurobehavioral outcomes reported induced by PAE. It would be more accurate also to include the references.

*- We apologize for this oversight and have added the appropriate reference (Mattson et al., 2019).*

• In the Results section titled “RNA-sequencing differentiates ethanol-fed from pair-fed rats”, it is reported the BEC collected during prenatal alcohol exposure, BEC range: 14.90 -110.70; mean: 44.03 ± 7.21. Unfortunately, it is not mentioned the unit used for the measurement both here and in the Material Methods Section. Furthermore, they are talking about moderate PAE, but usually, for a moderate model, the BEC is around 80-90 mg/dl. Please clarify this point.

- *We obtained blood samples from a separate set of dams given the ethanol diet. We have clarified the collection procedure and the diet procedure to address this point and others that the reviewer made. Blood samples were gathered at 8:00 pm (3 hours after the diet was placed on the cages). Using this time point, blood samples ranged from 14.90-110.70 mg/dL. A separate study using the same administration paradigm (Bodnar et al., 2021) gathered blood samples at 7:00 am and reported significantly higher BECs (122.2 ± 26 mg/dL). It is possible that we would have gotten higher BECs if blood samples were collected at the end of the dark cycle. We plan to adjust the timing of blood samples going forward to reflect this finding. We have added this information to the Materials and Methods.*
- *We do have data for the amount of diet consumed for each dam on each day. On average, ethanol dams consumed 89.0 ± 8.24 mL of the liquid diet, which would equate to 5.96 mL of ethanol (95%) in a 24-hour period. We can add this data into the manuscript if requested.*

• As mentioned previously, the PAE paradigm used is described in the Material and Methods section. The current version is not clear to readers, it would be better to add more details such as the age of female rats before starting with the paradigm, and the number of litters used. It seems that for each experimental group, the authors used one litter since it is reported in line 134 “ with the exception of the alcohol-fed litter where only two females were available”. Please clarify this point.

- *The females used for breeding were approximately 9 months of age, which has been added to the manuscript for clarification. It is correct that for tissue collection, offspring were derived from one litter per treatment group. We elected to limit litter variability and include males and females in the analysis. We realize that this is a limitation of the current study and have addressed this point in the discussion (paragraph inserted below for ease).*
- *“As with any study, there are limitations to the current data. First, we elected evaluate gene expression in the hippocampus only, while acknowledging that gene expression, and its changes following PAE are likely to be region-dependent. However, changes in gene expression of protocadherins following developmental exposure have been observed in mouse cortex [12], human buccal samples [16,37], and zebrafish embryo [38], suggesting that this may be a reliable consequence of prenatal alcohol exposure. Another important factor to consider is the timing of alcohol exposure. In the rodent, prenatal alcohol administration results in exposure that is limited to the first and second trimester periods. It is entirely possible that a different outcome would be observed with postnatal exposure during the brain growth spurt (PD 4-9). Future studies should evaluate whether the timing of exposure is an important factor in the current outcome. Finally, we were limited in the number of samples that could be analyzed in the current study. After finding no sex differences, we elected to combine males and females, resulting in n’s of 5-6/group (2-3 males and females) Further, these rats were derived from a single litter per treatment group, with the goal of minimizing litter variability in order to focus on treatment effects. We recognize this as a limitation of the current work and plan to replicate and expand these studies going forward. However, the current findings align well with the existing literature, providing support for the current data. Finally, future studies should validate the expression changes observed in the current study..”*

Also, the readers have to assume that the exposure to alcohol started during the first day of gestation (G1) until G20. Please add more details about the protocol. Any specific reason for choosing this period of time?

- *Yes, diet administration occurred throughout gestation. Diet administration started on G1 and continued to G20, when all rats were switched to standard food and water. This timeframe was chosen to encompass the first- and second-trimester equivalent periods of brain development. This point has been clarified in the revised manuscript.*

• Furthermore, it would be more accurate to report the concentration of Ethanol used in the experimental diet and the average consumption during 1.5-2 hours of exposure- drinking in the dark. Furthermore, it is not clear if the dams were exposed for 1.5 or 2 hours. Clarify this critical point.

- *This point has been clarified in the revised manuscript. This was not a drinking in the dark procedure. Instead, the liquid diet was available the entire time during gestation. Water was available ad libitum, but the liquid diet was the only source of food. This approach has been used before in the PAE field (e.g., Riley et al., 1979, Driscoll et al., 1980, Holman et al., 2018, Bodnar et al., 2021). See also Marquadt & Brigman (2016) for an excellent review of the behavioral consequences of developmental alcohol exposure in rodents that includes details about experimental paradigm.*
- *We do have data for the amount of diet consumed for each dam on each day. On average, ethanol dams consumed 89.0 ± 8.24 mL of the liquid diet, which would equate to 5.96 mL of ethanol (95%) throughout the 24-hour exposure period. We can add this data into the manuscript if requested.*

• In the same section, authors reported the analysis performed for comparing possible differences in the body weight of pups at P7, showing a significant difference between males and females. Add more details about the statistical test used for those comparisons.

*- Thank you for this suggestion and we apologize that the statistical analyses used were not described appropriately. We conducted an ANOVA to evaluate body weight at PD 14, 21, and at tissue collection and have added this information into the manuscript. Since we did not observe many differences, we chose to briefly mention this analysis and not focus on weight in the results section.*

- In the section “Hippocampus tissue collection”, the authors reported that the animals were decapitated without anesthesia. Why did they decide to use this procedure? The stress during the sacrifice could be affected the result. Do the authors consider the possible contamination of blood?

*- Prenatal alcohol exposure can result in significant changes to the HPA axis. Thus, we wanted to collect the tissue as quickly as possible, to avoid possible differences as a function of stress exposure. Tissue collection occurred quickly (within 3-4 minutes) upon removing the rat from the home cage. We have added a sentence to clarify this point in the Materials and Methods section.*

*- We cannot rule out the possible contamination of blood, and future studies may compare the current findings to gene expression in tissue that has been perfused with saline prior to collection.*

• In the discussion section, the authors compared their findings with the current literature talking about differences related to the brain region examined and sex differences. However, they did not mention the possible differences related to the prenatal alcohol exposure paradigm used (prenatal vs 3rd trimester), the alcohol concentration.

*- This is an excellent point and we have added this to the discussion (Lines 415-432 of the revised manuscript)*

Minor comments
1. The authors across the entire manuscript use the abbreviation FASD for Fetal Alcohol Spectrum Disorders as indicated in line 29, since they are using the plural of disorder, it would be more correct to add the “s” to the abbreviation FASD.

- *The standard abbreviation for Fetal Alcohol Spectrum Disorders is FASD (example reference: Mattson et al., 2019, May et al., 2009). The full term is already in plural form, so adding an ‘s’ to the abbreviation would not be appropriate.*

2. Please indicate the acronym for Fetal Alcohol Syndrome (FAS).

- *This has been corrected.*

3. Several times, the authors did not spell out the acronyms used, for example, it would be more appropriate to define MRI (line 74), DTI (line 76).

- *This has been corrected.*

4. In the Author Summary section, it is used the term mothers for the rat dams. It would be more appropriate to use the term dams.

- *This has been corrected.*

5. In line 67, the authors defined the acronym PAE for Prenatal Alcohol Exposure, but they did not use it in the entire manuscript. Please be consistent in using the acronyms. Lines: 81, 90, 100, 106, 109, 111, 114, 119, 122, 129, 249, 268, 274, 278, 280, 282, 308, 311, 313, 315, 320.

- *This has been corrected, with the exception that the full term still remains in subheadings and at the beginning of the introduction to remind readers of the abbreviation.*

6. When they are going to indicate the age of the animals, explain for the first time the meaning of P indicating postnatal day.

- *This has been corrected.*

7. Table 1: typo for “adhesion”

- *This has been corrected.*

Reviewer #2: In this manuscript, Amal Khalifa, et al. isolated RNA from the hippocampus of adolescent rats exposed to ethanol during prenatal development and compared gene expression to controls. They found many genes were downregulated and these genes were associated with cell adhesion, toxin metabolism, and immune responses. They concluded from this study that changes in genetic architecture after prenatal exposure to alcohol continue through adolescent development.
There are some concerns.

1. From fetus to adolescence, there are many factors that could affect the changes in gene expression. Even among littermates, gene expression can vary significantly. Therefore, in order to identify the effect of fetal alcohol exposure on adolescent gene expression, a relatively large sample size is required to eliminate the influence of these factors. In this study, three males and three females were sampled for each group, with the exception of the alcohol-fed group that only two females were sampled. When analysis, three outlier samples were removed. The results from a small sample size study are likely affected by many factors, resulting in false positive results.

*- We recognize that this is a limitation of the current study and have added this to the discussion. We elected to include both males and females in the analysis, but few sex differences were observed. Thus, we were able to combine the males and females in the analysis. In addition, we realize that there is variability even within litters, so we chose to limit our analysis to a single litter for each group, in order to avoid introducing litter effects into the analysis. Furthermore, we have added some references throughout the paper that also found changes in expression of protocadherins following prenatal alcohol exposure. We feel that this helps provide support from the literature for the current data. We have clarified these important points throughout the manuscript.*

2. For the most differentially expressed genes, the expression and function of these genes of interest need to be further validated.

*- We agree, and have included this as a point in the discussion. However, it is beyond the scope of the current manuscript.*

3. As for sex differences, in Table S5, the manuscript compared ET Males with ET Females. In Table S6, compared PF Males with PF Females. As readers and reviewers, we also hope to know the comparison between ET Males and PT Males and the comparison between ET Females and PT Females. I think the authors should perform the comparisons and include them into the manuscript, even though the principle components analysis (PCA) shows that sex is not a primary factor.

*- We thank the reviewer for the suggestion to include this information in the manuscript. We have now included two additional supplemental tables (Tables S10-11) comparing PF and ET male samples and female samples independently. We also provide a visual comparison of these lists in Venn diagrams to demonstrate the overlap between down and upregulated genes in all three analyses (Supplemental Figure 2). These data demonstrate that most of the genes identified in sex-specific analysis were also identified in the analysis on all compiled samples. Furthermore, there are only a small number of genes uniquely identified in male or female-only analysis, suggesting that biological sex has little impact on differential gene expression in this study. Furthermore, we see a substantial increase in significantly altered genes when male and female samples are compiled, demonstrating the increased power we gain by increasing biological replicates in this way. We now include these explanations in the text.*

4. On page 25, in the first line, in Figure 2B, the volcano plot was generated from data comparing ET rats to AD rats, not PF rats. This might be a typo.

*- We appreciate this comment. It was in fact a typo that has been corrected.*

6. PLOS authors have the option to publish the peer review history of their article ([what does this mean?](https://journals.plos.org/plosone/s/editorial-and-peer-review-process#loc-peer-review-history)). If published, this will include your full peer review and any attached files.

If you choose “no”, your identity will remain anonymous but your review may still be made public.

**Do you want your identity to be public for this peer review?** For information about this choice, including consent withdrawal, please see our [Privacy Policy](https://www.plos.org/privacy-policy).

Reviewer #1: No

Reviewer #2: No
